# Towards an Established Intraoperative Oncological Favorable Tool: Results of Fluorescein-Guided Resection from a Monocentric, Prospective Series of 93 Primary Glioblastoma Patients

**DOI:** 10.3390/jcm12010178

**Published:** 2022-12-26

**Authors:** Jacopo Falco, Emanuele Rubiu, Morgan Broggi, Mariangela Farinotti, Ignazio G. Vetrano, Marco Schiariti, Elena Anghileri, Marica Eoli, Bianca Pollo, Marco Moscatelli, Francesco Restelli, Elio Mazzapicchi, Emanuele La Corte, Giulio Bonomo, Marco Gemma, Giovanni Broggi, Paolo Ferroli, Francesco Acerbi

**Affiliations:** 1 Neurosurgical Unit 2, Department of Neurosurgery, Fondazione IRCCS Istituto Neurologico Carlo Besta, 20133 Milan, Italy; 2 Neuroepidemiology Unit, Scientific Directorate, Fondazione IRCCS Istituto Neurologico Carlo Besta, 20133 Milan, Italy; 3 Department of Biomedical Sciences for Health, University of Milan, 20122 Milan, Italy; 4 Unit of Molecular Neuro-Oncology, Fondazione IRCCS Istituto Neurologico Carlo Besta, 20133 Milan, Italy; 5 Neuropathology Unit, Fondazione IRCCS Istituto Neurologico Carlo Besta, 20133 Milan, Italy; 6 Department of Neuroradiology, Fondazione IRCCS Istituto Neurologico Carlo Besta, 20133 Milan, Italy; 7 Neurointensive Care Unit, Department of Neurosurgery, Fondazione IRCCS Istituto Neurologico Carlo Besta, 20133 Milan, Italy; 8 Experimental Microsurgical Laboratory, Department of Neurosurgery, Fondazione IRCCS Istituto Neurologico Carlo Besta, 20133 Milano, Italy

**Keywords:** glioblastoma, sodium fluorescein, neuro-oncology, YELLOW 560 filter, fluorescence-guided neurosurgery, gross total resection, overall survival

## Abstract

It is commonly reported that maximizing surgical resection of contrast-enhancing regions in patients with glioblastoma improves overall survival. Efforts to achieve an improved rate of resection have included several tools: among those, the recent widespread of fluorophores. Sodium fluorescein is an unspecific, vascular dye which tends to accumulate in areas with an altered blood–brain barrier. In this retrospective analysis of patients prospectively enrolled in the FLUOCERTUM study, we aimed to assess the role of fluorescein-guided surgery on surgical radicality, survival, and morbidity. A retrospective review based on 93 consecutively and prospectively enrolled IDH wild-type glioblastoma patients (2016–2022) was performed; fluorescence characteristics, rate of resection, clinical outcome, and survival were analyzed. No side effect related to fluorescein occurred; all of the tumors presented a strong yellow-green enhancement and fluorescein was judged fundamental in distinguishing tumors from viable tissue in all cases. Gross total resection was achieved in 77 cases out of 93 patients (82.8%). After a mean follow-up time of 17.4 months (3–78 months), the median progression-free survival was 12 months, with a PFS-6 and PFS-12 of 94.2% and 50%, respectively, whereas median overall survival was estimated to be 16 months; survival at 6, 12, and 24 months was 91.8%, 72.3%, and 30.1%, respectively. Based on these results, we can assert that the fluorescein-guided technique is a safe and valuable method for patients harboring a newly diagnosed, untreated glioblastoma.

## 1. Introduction

Brain malignancies account for approximately 2% of the tumors afflicting humankind [1]. Among malignant gliomas, glioblastoma (GBM) represents the most common and lethal form in adults [2]. Histologically, glioblastoma features prominent cellular and nuclear atypia, numerous mitotic figures, necrosis, and microvascular proliferation [3]. However, first, the WHO classification of central nervous system (CNS) tumors edited in 2016 [4] and, overall, the 2021 update (CNS5) have introduced relevant innovations in tumors’ definition, underlining the preponderant role of genetic elements as diagnostic criteria if compared to morphological ones [3,5]. Indeed, GBM diagnosis is appropriate for each IDH wild-type astrocytic tumor presenting with concurrent +7/−10, EGFR amplification, or a TERT promoter mutation, even in the absence of classical high-grade histopathologic features [6,7]. Radiologically [8], GBM usually presents in the form of a bulky mass with heterogeneous enhancement and central necrosis. Concomitantly surrounding T2/FLAIR abnormality indicates areas of edematous, infiltrating, and non-enhancing neoplastic tissue.

Despite decades of research on its pathobiology and clinical management, GBM still possesses an ominous prognosis, with a median survival of 15–18 months [2,9]. Maximal safe resection followed by radiation therapy with concurrent and adjuvant temozolomide, as described by Stupp in 2005 [9], still represents the standard of care. The first act of the therapeutic play, namely surgery, varies from a minimally invasive biopsy to gross total resection (GTR). Even when GTR is achieved and supplemented with systemic therapies, the patient ultimately succumbs to the disease [10]. Hence the need to highlight the factors related to a rather better prognosis arises. Among those factors [11], the extent of resection (EOR) has been linked to an increase in overall survival (OS) and progression-free survival (PFS) [12]. However, no consensus exists regarding the optimal EOR in improving patients’ prognosis [13]. Moreover, GTR, i.e., the complete resection of the contrast-enhancing (c.e.) portion of the lesion, has been reported to be achievable only in a small percentage of cases [14,15]. This difficulty is mostly related to the identification of tumor margins [16] and appears to be scarcely affected by the introduction of technological tools such as neuronavigation [17], intraoperative brain MRI [18], and ultrasound [19].

Therefore, identifying different adjuncts to guide resection has become a tactical challenge in achieving maximal safe tumor resection. This need has fostered the exploration of a relatively new realm, that of fluorophores [20] such as 5-aminolevulinic acid (5-ALA) and sodium fluorescein (SF). 5-ALA is a natural precursor of hemoglobin that prompts the metabolic synthesis of fluorescent porphyrins in neoplastic cells [21]. Its application in malignant glioma resection has increased the EOR (65% of GTR in the experimental group vs. 36% of patients randomized to the white light illumination group in the trial published by Stummer and coworkers in 2006), enabling for a consequent improvement in PFS at 6 months [16,22,23]. Despite displaying high specificity for neoplastic cells, 5-ALA’s widespread use in tumor margin resection is curtailed by several factors, such as the need to administer the drug orally 3 h before the induction of anesthesia and to avoid sunlight skin exposure for 24 h after surgery due to the photosensitizing effect [24]. Furthermore, although it has an outstanding role in increasing the rate of tumor resection as demonstrated by means of a phase III study, the achieved GTR in 65% of patients must be further improved.

On the other side of the tumor visualization battlefield, SF is encountered. This dye emits fluorescent radiation in the wavelength ranging from 540 to 690 nm and builds up in cerebral areas presenting damage to the blood–brain barrier (BBB) [25,26]. The implementation of dedicated filters in the surgical microscope has improved the discrimination of normal and neoplastic brain tissues, reducing the requested drug dose to obtain this effect [14,27]. Although less specific compared to 5-ALA, SF’s advantages are testified by its intravenously administration during the induction of anesthesia, its lower costs, and by the extremely low incidence of side effects [28], guaranteeing GTR in more than 80% of fluorescent cases in our multicentric phase II study. Furthermore, when comparing the intraoperative fluorescein staining of the tumor with the preoperative MRI, the role of SF extends to that of a valid tracer for abnormal tissue in both c.e. and non-c.e. regions of high-grade gliomas (HGG) [29,30,31]. It is not entirely understood why this occurs, although the proposed mechanism includes different degrees of vascular disruption between c.e. and non-c.e. regions, discrepancies in the vascular permeability of fluorescein and gadolinium, and altered patterns of diffusion within the extracellular space [32].

In recent years, also thanks to our preliminary studies and the approval, in July 2015 (determination 905/2015), of the fluorescein molecule as a neuro-oncological tracer by the Italian Medicine Agency (AIFA), SF applications have increased exponentially [14,25,33,34]. According to AIFA determination, the intravenous (i.v.) injection of SF as a neurosurgical tracer during neuro-oncological procedures has been approved and its cost is totally reimbursed by the Italian national health system. A low dose (5 mg/kg) of fluorescein is i.v.-administrated at the end of patient intubation [35]. In March 2016, the authors started a new prospective observational study, called FLUOCERTUM (FLUOrescein in CERebral TUMors), regarding the use of SF as a fluorescent intraoperative tracer in patients with c.e. tumors of the CNS, both in adult and pediatric patients [26,34,36,37,38].

Therefore, considering that residual neoplastic tissue extending beyond c.e. borders contributes to disease recurrence, we hypothesize the potential role of SF in the implementation of a supramaximal GBM resection. In this study, we retrospectively analyzed data from the FLUOCERTUM prospective study conducted at our institute to investigate the utility of SF in improving the rate of GTR of GBM and to assess the outcomes of patients undergoing fluorescein-guided resection.

## 2. Materials and Methods

### 2.1. Patients

In this study, we retrospectively reviewed the database of the prospective observational FLUOCERTUM study, started in March 2016 and approved by the institutional review board, to identify the cohort of GBM patients until June 2022. Written informed consent for the surgical procedure, including the use of sodium fluorescein, as well as to participate in this observational study was obtained from each patient. The protocol and the retrospective case series revision have been approved by the Ethical Committee of the Fondazione IRCCS Istituto Neurologico Carlo Besta.

The inclusion criteria of the FLUOCERTUM study were as follows: patients of both genders, of any age, with suspected aggressive lesions of the CNS, as suggested by preoperative MRI with i.v. contrast agent administration. The exclusion criteria were: impossibility to give consent due to cognitive deficits or language disorders; a known allergy to contrast agents or history of previous anaphylactic shock; known severe previous adverse reactions to SF; acute myocardial infarction or stroke in the last 90 days; severe organ failure; and women in their first trimester of pregnancy or lactation [26].

The retrospective case series revision aims to identify those patients scheduled for fluorescein-guided surgery with a histopathologically-confirmed GBM according to the 2021 WHO CNS5 tumor classification [5]. Indeed, the study population was limited to patients with newly diagnosed, untreated GBM (i.e., the IDH wild-type GBM according to the 2016 classification) considered suitable for surgical removal; in particular, tumors with a large, non-c.e. area, suggesting low-grade gliomas with malignant transformation and multicentric lesions were excluded. Otherwise, multifocal tumors suitable for radical resection by means of a single surgical approach were included; only patients in which total fluorescein enhancement was removed were considered in the statistical analysis for the aims of this study. In our experience, which was the regular way of completion even in this case series, no aprioristic volumetric cut-off or morphological features were considered as an absolute contraindication to surgery, and we always pushed far beyond the conventional limits of surgical indications in cases of patients with a good performance status, no comorbidities, and a high motivation to go through a complex process of care. No limitation of the Karnofsky performance scale (KPS) were considered for outcome analysis [39].

### 2.2. Pre- and Postoperative Clinical and Radiological Evaluation

The preoperative assessment included physical and neurological examination (KPS), laboratory tests results, and contrast-enhanced MRI for neuronavigation. If judged necessary, fMRI and DTI were acquired for the best surgical planning. All of the patients were submitted to MRI, including 3D volumetric sequences with and without contrast (T1 weighted); tumor volume was calculated [40] by segmentation of the whole contrast enhancement (Osirix for Macintosh, www.osirix-viewer.com, accessed on 10 October 2022) by a dedicated neuroradiologist (M.M.). No aprioristic distinction was performed among tumors located in non-eloquent brain areas or adjacent to them (motor/sensory cortex, visual area, speech center, internal capsule, basal ganglia, hypothalamus/thalamus, brainstem, and dentate nucleus); as affirmed by the abovementioned information, only once complete fluorescent tissue removal was achieved was the case considered for further analyses.

To evaluate the EOR, a volumetric MRI examination was performed for each patient within 72 h of surgery; in particular, to calculate the residual pathological residual volume (RV), the hyperintense alterations in volumetric basal T1 acquisitions were subtracted from the volume of hyperintense tissue in post-contrast volumetric T1 images to avoid the incidental inclusion of blood or blood products [41]. The EOR was calculated as a percentage of tumor resection based on early contrast-enhanced postoperative MRI.

Surgery was to be followed by standard conformational radiotherapy and concurrent chemotherapy with temozolomide, followed by six cycles of maintenance chemotherapy with temozolomide according to the Stupp protocol [9,42]. No restrictions were imposed on treatment after disease progression [43].

The postoperative clinical evaluation included a standard neurological examination as above, including KPS evaluation, as well as laboratory tests (kidney function) and exclusion of the occurrence of any side effect related to fluorescein injection. Periodic MRIs were performed according to regular clinical practice; the first clinical and radiologic evaluation following the postoperative one was performed before the beginning of radiotherapy and concomitant temozolomide; subsequently, follow-up was performed every two cycles of temozolomide during the Stupp protocol phase and, after tumor progression and change in chemotherapeutic approach, every 2 months. The demographics, clinical, neuroimaging, surgical, pathological, and adjuvant treatments data were obtained from the personal medical records, the institutional cancer registry, the institutional complication registry, the institutional pathology registry, follow-up visits, and telephonic interviews.

### 2.3. Surgical Protocol and Intraoperative Fluorescence Characterization

The standardized surgical protocol of the fluorescein-guided technique, as already described in previous papers, is based on i.v. SF (Monico S.p.A., Venice, Italy) injection at a standard dose of 5mg/kg, by a central or peripheral venous line, immediately upon completion of the induction of general anesthesia or anesthetic procedures in awake surgery [35].

Surgery was performed with the aid of a surgical microscope equipped with an integrated fluorescent filter tailored to the excitation and emission wavelength of sodium fluorescein (YELLOW 560—Pentero 900 or Kinevo microscopes; Carl Zeiss Meditec, Oberkochen, Germany). During resection, the microscope could be switched alternatively from fluorescent to white light illumination; neuronavigation (Stealth S7 or S8; Medtronic Inc. Minneapolis, MN, USA), intraoperative ultrasounds (MyLab; Esaote, Genoa, Italy), or other tools could be used according to the surgeon’s preference [44,45]. The tumors were removed in an inside-out fashion until all fluorescent tissue was removed, as considered feasible by the surgeon [26]. Surgery was pushed toward the radicality, starting with an intralesional approach, especially in patients without comorbidities and a favorable clinical status, that can maximally take advantage of this clinical behavior.

At the same time, to allow the safest surgery, in tumors located adjacent to eloquent areas, intraoperative neurophysiological monitoring (IONM) was used [46]; awake anesthesia was considered in specific patients, especially in cases of primary language regions involvement, after an accurate neuropsychological workshop. In cases where tumor vessels or peritumoral arteries and veins needed to be intraoperatively evaluated, indocyanine green (ICG) video-angiography with FLOW 800 analysis (Carl Zeiss Meditec, Oberkochen, Germany) and, sometimes, ICG temporary clipping test were performed, as already reported [47,48,49].

The surgical procedures were performed by four different neurosurgeons (M.B., M.S., P.F., and F.A.) with significant experience in the neuro-oncological field, after an adequate learning curve in using SF, since its first application at our institution in 2011 [50]. The fluorescence intensity was graded by the surgeon as intense, inhomogeneous or homogeneous, moderate, slight or absent; the surgeons were also asked to classify the use of SF per procedure as helpful, partially helpful, unhelpful, or not essential to achieve the surgical aims.

### 2.4. Histological Analysis and MGMT/IDH Determination

Histopathological analysis was performed in each case (B.P.); the tumors were classified according to the 2016 or 2021 WHO classification by the neuro-pathology group of our institute. IDH mutation was evaluated by immunohistochemistry; only glioblastoma patients as standardized according to CNS5 were included in this study, i.e., GBM IDH-wt.

The methylation patterns in the CpG islands of the O-6-methylguanine-DNA methyltransferase (MGMT) promoter were determined by chemical modification of unmethylated cytosines to uracils, followed by methylation-specific PCR [51].

### 2.5. Endopoints and Study Aims

The principal aim of the present study is to assess the contribution and the valuable role of fluorescein in the surgical resection (rate of GTR and RV) and visualization (fluorescein usefulness) of primary, untreated glioblastomas with a dedicated filter integrated in the surgical microscope.

Secondary endpoints consist of evaluating short- and long-term clinical outcome, PFS at 6 and 12 months, and OS.

Disease progression was defined as evidence of a new measurable enhancing lesion for patients with total removal or a ≥25% increase in the sum of the products of perpendicular diameters of enhancing lesions compared with the smallest tumor measurement at baseline for patients with incomplete removal, according to the RANO criteria [52]. PFS-6 and PFS-12 were defined as the proportion of patients with no progression at 6 and 12 months after diagnosis, respectively. Patients who died from any cause before documented progression were counted as an event for this endpoint. The OS was defined as the number of patients who had not died from any cause.

### 2.6. Statistical Analysis

The sample was described by means of the usual descriptive statistics: the mean, median, and standard deviation for continuous variables and the proportions for categorical ones. PFS and OS were estimated by the Kaplan–Meier method; PRISM software for Macintosh was used for the statistical analysis.

## 3. Results

**Figure 1:**
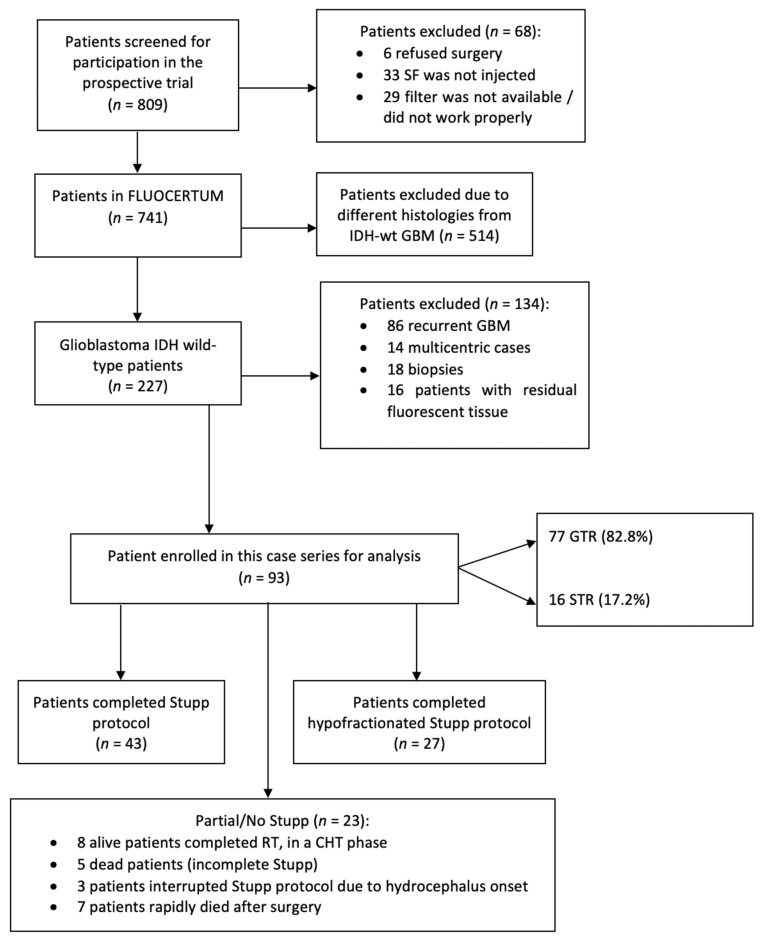
Flow diagram of patients’ enrollment and management.

Ninety-three patients met the inclusion and exclusion criteria: patients’ ages ranged from 30 to 82 years, with a mean age of 61.2; 61 males (65.6%) and 32 females (34.4%) were enrolled in the study (Figure 1).

The mean preoperative tumor volume was 60.27 cm^3^ (range: 0.74–334.69 cm^3^). All of the patients presented with a heterogeneous c.e. on the preoperative MRI, with different degrees of intensity, and, frequently, cystic-necrotic components, especially at the lesion core. Most patients underwent surgery until 2021; the cases have been reviewed to identify only glioblastoma IDH wild-type patients (GBM, grade 4, according to 2021 CNS5 tumor classification); in 53 out of 93 cases (57%), the MGMT promoter was methylated whereas it was unmethylated in the remaining 40 patients (43%).

The majority of the tumors were located in not-eloquent regions (57 cases out of 93–61.3%); otherwise, 30 patients (32.2%) were scheduled for IONM-assisted surgery, whereas in six cases (6.5%), an awake anesthesia was planned.

A summary of the demographic, surgical, and outcome characteristics of the patients is presented in Table 1.

### 3.1. Intraoperative Fluorescence Characteristics and SF Effects in EOR

No technical difficulties regarding the use of the microscope filter nor switching between white and yellow light were encountered during the surgical resections. No adverse drug reaction related to SF injection was reported in this cohort of patients; the only remarkable and visible effect was the transient yellowish staining of urine, which disappeared in about 24 h.

**Figure 2:**
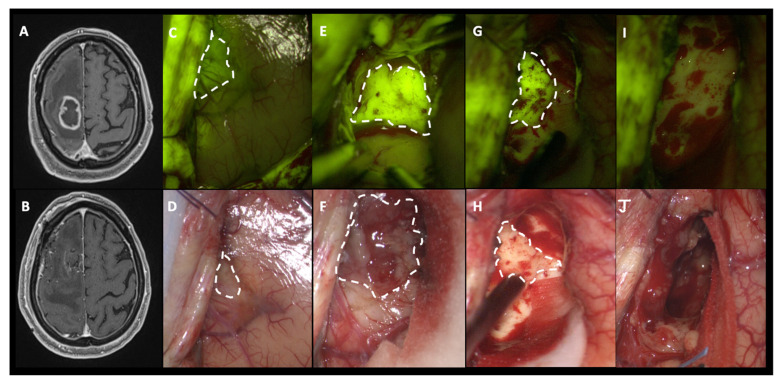
(**A**) Preoperative post-contrast axial T1-weighted MRI scan showing a right frontal GBM that was completely removed, as visible in post-operative T1 with a contrast axial scan (**B**). Intraoperative images, under YELLOW 560 filter (**C**,**E**,**G**), depict a bright yellow-green signal (dotted lines) in correspondence to the pathological components; the corresponding images are shown and compared under white light visualization (dotted lines in (**D**,**F**,**H**)): at the beginning of surgery (**D**), it is possible to appreciate a grayish, vanishing area which corresponds to the cortical projection of pathological tissue but during surgical resection, fluorescein becomes fundamental in helping the surgeon to identify residual pathological tissue (dotted line in (**G**)), not clearly visible under white light (**H**). (**I**,**J**) At the end of the resection, the cavity did not show any sign of residual tumor, as confirmed by fluorescent visualization with the YELLOW 560 filter.

All tumors included in this study showed a highly intense fluorescent yellow-green signal, clearly distinguishable from peritumoral brain parenchyma, depicted as pinkish tissue (Figure 2 and Supplementary Materials Video S1). Each case presented specific difference in the pattern of fluorescence enhancement which could be more heterogeneous due to the presence of darker necrotic areas. In addition, in patients with large cystic components, the cystic fluid typically appeared brightly fluorescent as well. It is well known that specific intracranial areas, due to the absence of BBB, could result as fluorescent tissue such as dura mater. In all of the procedures, intraoperative fluorescence was deemed helpful in achieving a complete resection by means of a better delineation of the borders of the tumor tissue from the healthy parenchyma as compared with the conventional microsurgical technique using white light illumination. Fluorescence enhancement was predominantly intense and inhomogeneous, tracing the c.e. of the preoperative MRI; in few cases, especially in small and compact lesions, SF uptake can appear homogeneous in contrast to large and necrotic gliomas with a more heterogenous enhancement. Despite these specific peculiarities, no differences among efficient SF visualization and tumor removal were detected during surgery nor was a different rate of surgical radicality appreciated.

**Figure 3:**
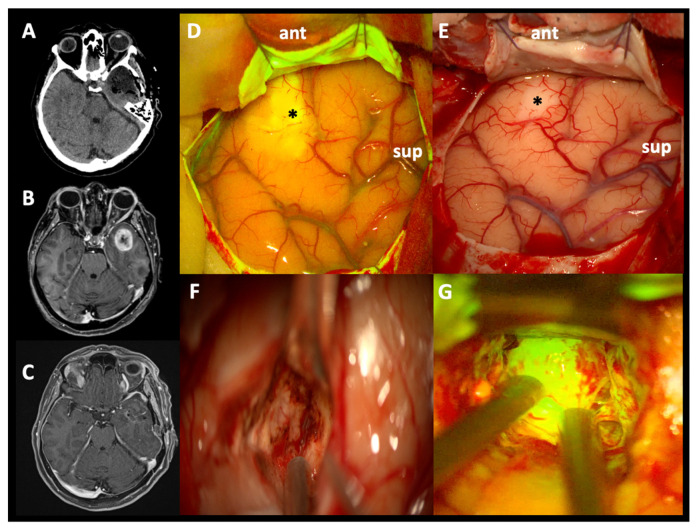
(**B**) Preoperative post-contrast axial T1-weighted MRI scan showing a left temporal GBM, that was completely removed, as visible in post-operative T1 with a contrast axial scan (**C**). A left temporal approach was performed; the craniotomy was executed by means of a basal burr hole and craniotome, as visible in the postoperative axial CT scan (**A**). Intraoperatively, after dural opening with anterior hinge, it is possible to appreciate both under with light (asterisk in (**E**)) and after YELLOW 560 filter activation (asterisk in (**D**)) a small temporobasal contusion which appeared brightly fluorescent. Despite this eventuality, the YELLOW 560 filter maintained its utility in tumor recognition (**G**), highlighting pathological fluorescent tissue when compared to white light visualization (**F**).

We have never found SF uptake during surgical manipulation of healthy tissue in peritumoral parenchyma because of the limited amount of circulating fluorescein due to our strict protocol of low-dosage fluorescein use, thanks to the specific YELLOW 560 filter, injected at least 1 h before dural opening [35]. However, it is possible to find some examples of unspecific fluorescein enhancement in cortical areas far from the tumor tissue, due to minimal contusion happening in the first phases of surgery (i.e., burr holes), closer to fluorescein i.v. injection (Figure 3), when a significant amount of fluorescein molecules is still present in the blood circulation [25]. Furthermore, minimal fluorescence can be visible in close sulci and around cortical veins, due to extravasation of fluorescent liquid in the subarachnoid space [14]. This incidental event has been observed in eight patients of this cohort (8.6%), without compromising the success of the surgery; this finding is in line with our experience with a nonspecific fluorescence rate of less than 10% of cases.

GTR was achieved in 77 cases out of 93 patients (82.8%); the remaining 16 patients (17.2%) with a subtotal tumor resection had a mean RV of 4.05 cm^3^ (0.17–22.76 cm^3^) or 4% of the initial tumor mass (0.6–9.7%). Six out of 16 cases (37.5%) of incomplete removal [41,53] had a >98% EOR (Table 1).

In few cases of large and necrotic tumors, the RV can be considered high in the absolute sense, although less than 10%; we have interpreted this eventuality to be due to the prolonged surgery, which causes a higher wash-out of fluorescein from the blood circulation and the hemorrhagic feature of these lesions, that can further reduce the capacity of SF discrimination attenuating the contrast between fluorescent and not-fluorescent tissue. Due to the small number of patients harboring a large tumor, we are not able to perform a statistical inference to demonstrate our empirical considerations.

### 3.2. Clinical Outcome, PFS, and OS

At baseline, 54/93 (58.1%) of patients had a KPS score of 90–100, indicating excellent clinical and neurological conditions, 33/93 (35.5%) of patients had a KPS score of 70–80, indicating moderate clinical and neurological conditions, and only 6 patients (6.4%) had a worse clinical and neurological state. Surgical morbidity led to a postoperative decline in KPS score in 29 patients at discharge, but only in 3 cases was clinical worsening marked with a decrease greater than 30 points in KPS score; 49 patients were discharged with an unchanged KPS score, whereas 15 patients presented with a clinical improvement using surgical treatment. At discharge, 51/93 (54.8%) of patients had a KPS score of 90–100, 29/93 (31.2%) of patients had a KPS score of 70–80, and 13/93 (14%) of patients had a KPS score ≤60 (Table 1).

**Figure 4:**
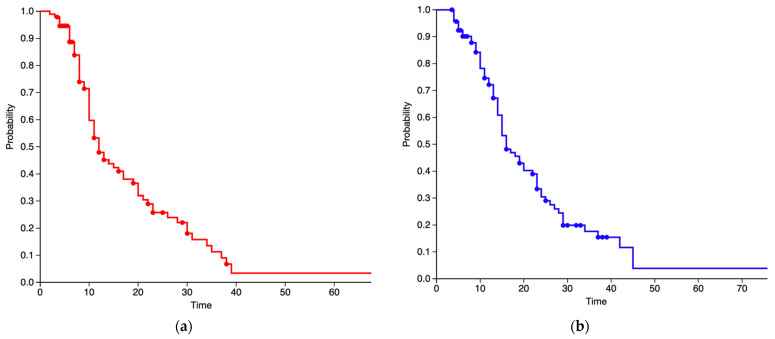
Kaplan–Meier curve of PFS (**a**) and OS (**b**). Time interval is depicted in months.

Forty-three patients (46.2%) completed the total Stupp protocol [9]; eight alive patients (8.6%) have completed concomitant radiotherapy and chemotherapy but are in different cycles of temozolomide. Five (5.4%) performed an incomplete Stupp protocol, including concomitant radiotherapy and temozolomide, but not all the six cycles of chemotherapy after the completion of radiotherapy. Three patients (3.2%) prematurely stopped the Stupp protocol after commencement due to acute hydrocephalus needing surgical management. Twenty-seven patients (29%) underwent hypofractionated radiotherapy (40 Gy in 15 fractions) and concomitant temozolomide due to age >65 years [42]. Seven patients (7.5%) did not undergo any postoperative treatment, due to early exitus, significant neurological deterioration, or extreme rapidly tumor progression (Figure 1). After a mean follow-up time of 17.4 months (3–78 months), the median PFS was 12 months, with a PFS-6 and PFS-12 of 94.2% and 50%, respectively (Figure 4a).

During the study period, 69 patients died due to tumor progression or other causes. The median OS was estimated as 16 months. Survival at 6, 12, and 24 months was 91.8%, 72.3%, and 30.1%, respectively (Figure 4b).

## 4. Discussion

It is well known that increasing the rate of HGG resection, achieving the GTR, improves survival since surgery is the mainstay of treatment with a propaedeutic role for adjuvant therapies according to the Stupp protocol [2,9,24].

For more than 10 years, SF has emerged as an intraoperative dye able to improve brain–tumor discrimination, due to its non-specific, vascular mechanism of action related to the accumulation in brain regions with BBB damage, as it happens with MRI c.e. in most aggressive, intra-axial tumors [8,54,55,56].

This study confirmed that the fluorescein-guided resection of GBM is a feasible, safe, and valuable intraoperative tool which allows for a high rate of complete resection at early postoperative MRI [25]. In all cases, fluorescein was judged helpful in tumor identification and discrimination from the peritumoral, sometimes edematous, brain parenchyma [26].

Our results showed a complete resection in 82.8% of the cases with a mean RV in the remaining 16 cases of 4.05 cm^3^; EOR of >98% was achieved in 89.2% of patients. Thus, the fluorescein-guided technique seems to be able to assure a high-rate of total or near-total resection in GBM. Even more important from a prognostic point of view, it should be noted that 14 patients out of the 16 incomplete resected cases (87.5%) showed an RV that was inferior to 5 cm^3^, with 64.2% of them (9 of 14 patients) being <2 cm^3^ [41,53]. Several recent studies have underlined an incremental survival benefit not only with a more aggressive surgical resection but also with patient characteristics such as age, which is a non-modifiable risk factor, and performance status due to the intrinsic correlation with tolerance and quick programming of adjuvant therapies. In this case series, about 90% of patients had a KPS score ≥70, suggesting a good postoperative clinical and neurological status. After a mean follow-up time of more than 17 months, the patients enrolled in this series presented a median PFS of 12 months and a median OS of 16 months. Although the experimental design of this study was not designed to determine survival data, the proposed results suggest an improvement of clinical outcome in fluorescent patients. Nevertheless, it is well known that MGMT methylation signature has a relevant effect on disease outcome [57,58] and in our cohort of cases just over 50% of patients had a methylated promoter; this incidental evidence could have minimally increased the survival data. At the same time, however, it should be considered as a counterpart that only less than half of the patients completed the full Stupp protocol. Unfortunately, no subgroups analysis could have been performed due to the design of the study and because of the small clusters of specific patients.

Glioblastoma still remains the most aggressive CNS tumor with a poor prognosis in the short term [1,59]; the WHO CNS5 classification has shrunk the molecular and morphological features to determine an adult-type diffuse glioma as a glioblastoma [5,7]. The modern forms of classification are paving the way to the development of tailored and targeted patient-specific therapies [60]; nevertheless, in the current situation, we must continue along the same therapeutic path started in 2005 from Stupp and coworkers [9]. Due to the extensive and wide infiltration of neoplastic cells into the host brain tissue in diffuse gliomas, it is impossible to think about a radical excision of HGG; the mainstay of treatment is therefore based on the complete resection of c.e. areas, if possible, without a functional impact, followed by radiant and chemotherapeutic adjuvant protocols. Therefore, the philosophy of maximal safe resection must always be kept in mind, consisting of eventually stopping resection to preserve neurological function, reduce the morbidity burden, and guarantee a good postoperative performance status allowing the prosecution of adjuvant therapies; i.e., aiming to achieve maximal survival benefit with a minimal risk of functional deficit. With this standard of care, the median survival is expected to be between 15 and 18 months with a PFS of approximately 7–8 months [61,62].

Surgical resection of HGG under white light illumination is a ruinous technique; in the control group of the pivotal randomized clinical trial about 5-ALA published by the group of Stummer, the rate of patients with complete c.e. areas removal was only 36% [16]. The awareness of the intrinsic difficulties in recognized tumoral borders under white light visualization has determined the flourishing of many intraoperative tools aiming to increase surgical radicality, such as neuronavigation, intraoperative MRI, c.e. ultrasound, and fluorophores. The current development of specific filters on the operative microscopes has recently contributed to the widespread diffusion of fluorophores as intraoperative contrast agents during aggressive tumor resection [14].

5-ALA, the biochemical precursor of hemoglobin that provokes the synthesis and accumulation of fluorescent porphyrins in several malignant cancers among which HGG, can be visualized by means of the BLUE 400 filter with specific wavelength of 620–710 nm after the excitation wavelength of 400–410 nm. 5-ALA has been approved both by the EMA and by the FDA as a neurooncological tracer for malignant gliomas and this approval has paved the way to its broad use. In 2006, Stummer et al. [16] published their trial regarding this molecule finding an increase of c.e. total tumor resection (65% vs. 36% of patients randomized to the white light illumination group) with an improvement in PFS. After the first enthusiastic phase of 5-ALA usage [63], several downsides of this tool have emerged including its high cost, the need for oral administration of the drug hours before anesthesia which excludes its applicability in an urgent setting, and the necessity for patients to avoid direct exposure to sunlight or strong room light for 24 h after surgery due to the risk of skin sensitization [64].

On the other side, fluorescein sodium salt [65], although with an initial exclusive use in ophthalmology with a very low rate of adverse events [66], was sporadically and anecdotally used in neurosurgery in the last century [14]. SF represents a water-soluble dye with a major blue excitation peak in the region of 460–500 nm and a major green emission peak in the region of 540 to 690 nm. Since the introduction of the YELLOW 560 filter, SF applications in neurosurgery have increased exponentially; fluorescein, differently to 5-ALA, could be considered as a vascular fluorophore and its use as a fluorescent tracer in neuro-oncological procedures relies on its accumulation in the extracellular space of the tumor, related to the passage through a ruptured BBB, characterized by vessels with increased permeability, just for the presence of high-density glial cells [26,29]. In our multicentric phase II trial, we found a total c.e. tumor resection in 82.6% of cases with a median RV of 0.19 cm^3^, which was calculated as every mismatch between the T1 post contrast administration sequence and T1 acquisition, without considering any minimal cut-off as non-specific enhancement [25]. Fluorescein is a readily available tracer, inexpensive, completely excreted after 24 h, and with a high profile of safeness. Furthermore, fluorescein-guided surgery can be performed in yellow light since this fluorescence module allows for a visualization of non-fluorescent tissue in natural color, guaranteeing a correct anatomical depiction and the possibility of performing ordinary hemostasis with the filter activated [28]. Finally, SF has a strong predictor of its appearance in the microscopic yellow view which is the preoperative MRI c.e.; this correlation has widened its field of applicability allowing for the flattening of learning curves due to the large number of tumoral entities which can benefit from its usage [29]. Thus, it is not surprising that an increasing number of neurosurgeons are utilizing sodium fluorescein as an adjunct in glioma resection.

Although the wider neuro-oncological field of application of SF if compared to 5-ALA, the most important application of fluorescein remains its use in improving HGG visualization and resection. In a study by Katsevman et al. [24], fluorescein-resected GBM was compared to a similar group of patients scheduled for tumor removal under white light, in a retrospective design study; the authors found that near-total resection was achieved in 73% of fluorescein cases vs. 53% in the non–fluorescent group, with an impact in median survival that was improved in the experimental group (78 weeks vs. 60 weeks). Wang et al. [32] purposed, as a future direction, the evaluability of SF in achieving a supramarginal GBM resection due to the known properties of fluorescein to accumulate even in peritumoral regions without c.e. where a subclinical altered BBB allows for the diffusion of SF. Recently, several studies have retrospectively compared the two molecules with intraoperative MRI in HGG resection; Naik et al. [67] concluded that these techniques did not statistically differ among them in achieving a GTR in HGG resection although a favorable trend was detected for the MRI, followed by SF, and, last, 5-ALA. Finally, another retrospective study by the group of Garbossa [68] asserts that 5-ALA and SF are equally useful in achieving GTR of the c.e. portion of tumor volume; the authors further analyzed the combination of both dyes in a small subgroup of patients revealing an advantageous trend in GTR and OS with the concomitant use of both fluorophores.

To our knowledge, this study is one of the largest case series to date demonstrating the beneficial effect of applying SF as an adjunctive tool in GBM surgery. By employing fluorescein, we were able to achieve a great number of GTRs. It is important to consider that, even with fluorescein-guided techniques as well as with any fluorophore, a complete removal of pathological tissue is not always possible; indeed, yellow contrast can be appreciated if the tumor is properly exposed during the surgical approach [25]; thus, some residual tissue may be left behind, especially when small corticectomy has been performed and the fluorescent tissue is in the blind corner of the approach. Patients undergoing fluorescein-guided surgery had an improved profile of outcome with prolonged PFS and OS. Several studies have been performed to analyze the sensitivity and specificity of SF in detecting malignant glioma [69]; in this case series, we did not systematically analyze this topic. As well, the accuracy in tumor tissue identification is further strengthened by the slight deterioration in KPS in a small group of patients with a marked decrease in performance status in only three cases, compared to the high number of patients harboring an eloquent tumor [70,71].

The main limitation of this study is represented by the lack of a direct comparison of surgery with and without SF aid, due to ethical reasons, or between different fluorophores (SF vs. 5-ALA), due to the authors’ surgical experience in the fluorescein field. Additionally, the patients were not centralized for oncological adjuvant therapies and this element could have influenced the survival analysis.

Despite these weaknesses, our study could represent another cornerstone that may indicate that fluorescein use is useful in the identification of tumoral tissue and in achieving a high rate of GTR of GBM. Further studies could better elucidate the contribution of fluorescein-guided techniques in improving the survival of patients harboring a malignant glioma. We also have to stress that, in most countries, SF is still considered off-label for neuro-oncological procedures. Thus, a widespread utilization of fluorescence-guided surgery will depend on definitive approval by the competent authorities.

## 5. Conclusions

This work corroborates sodium fluorescein as a promising, easy-to-apply, and affordable strategy for improving rates of total resection in patients affected by GBM with a possible impact on survival. Larger comparative studies, even including randomized clinical trials, will probably assess the advantage of this technique in terms of overall survival.

## Figures and Tables

**Table 1:** Summary of patient demographics, resection data, and survival.

Nr.	Sex	Age (years)	Location ^1^	KPS in	Vol. (cm^3^)	IONM	Histology	MGMT	Resection	RV cm^3^	KPS Out	OS (Months)
1	M	61	R parietal	90	5.38	yes	GBM	methylated	100%	0	90	26
2	M	82	R parietal	90	0.74	yes	GBM	methylated	100%	0	90	5
3	M	53	R frontal	90	43.01	no	GBM	methylated	100%	0	90	14
4	M	66	R parietal	90	89.65	no	GBM	methylated	100%	0	90	45
5	M	66	L temporal	90	21.57	yes	GBM	methylated	100%	0	90	15
6	M	51	R parietal	100	19.09	no	GBM	unmethylated	100%	0	100	42
7	M	71	R temporal	80	54.36	no	GBM	methylated	100%	0	90	78
8	M	81	L frontal	90	7.61	no	GBM	methylated	100%	0	90	10
9	M	62	R fronto-temporal	90	0.88	no	GBM	methylated	100%	0	90	22
10	F	70	L temporal	90	3.41	yes	GBM	methylated	100%	0	90	28
11	M	53	R parietal	90	92.53	yes	GBM	methylated	100%	0	70	34
12	M	49	L parietal	90	120.24	no	GBM	methylated	100%	0	90	23
13	M	66	L temporal	80	15.69	no	GBM	methylated	100%	0	70	4
14	M	62	L frontal	100	25.04	no	GBM	unmethylated	100%	0	100	29
15	M	65	L temporal	90	219.53	no	GBM	unmethylated	100%	0	90	10
16	F	59	L parietal	90	44.25	yes	GBM	methylated	100%	0	90	16
17	F	54	L temporal	60	39.79	yes	GBM	methylated	100%	0	40	15
18	F	51	L parietal	100	1.74	no	GBM	unmethylated	100%	0	100	11
19	M	48	R parietal	90	15.83	yes	GBM	unmethylated	100%	0	60	4
20	M	72	R temporo-parietal	90	117.64	yes	GBM	methylated	100%	0	90	12
21	M	45	L parietal	90	6.28	awake	GBM	unmethylated	100%	0	80	45
22	M	69	L temporal	90	16.84	no	GBM	unmethylated	100%	0	90	19
23	F	75	L parietal	80	31.69	yes	GBM	methylated	100%	0	60	12
24	F	47	R parietal	90	21.49	awake	GBM	methylated	100%	0	90	23
25	F	74	L fronto-parietal	90	7.74	no	GBM	methylated	100%	0	70	6
26	F	73	R parietal	80	16.89	yes	GBM	unmethylated	100%	0	80	4
27	M	73	R frontal	90	122.76	awake	GBM	unmethylated	99.4%	0.71	40	9
28	F	69	L parietal	90	4.95	no	GBM	methylated	100%	0	90	23
29	M	57	R frontal	90	48.89	yes	GBM	methylated	100%	0	90	37
30	F	60	R parieto-occipital	90	21.16	no	GBM	methylated	90.4%	2.04	80	17
31	M	45	R frontal	90	37.25	no	GBM	unmethylated	98.4%	0.59	100	8
32	M	75	L frontal	60	99.21	no	GBM	methylated	100%	0	50	13
33	F	64	L temporal	90	26.71	no	GBM	unmethylated	100%	0	90	20
34	M	76	L frontal	90	82.97	no	GBM	unmethylated	100%	0	90	5
35	M	62	L occipital	90	126.91	no	GBM	unmethylated	100%	0	70	24
36	M	47	R temporal	100	73.98	no	GBM	unmethylated	100%	0	100	24
37	F	72	R temporal	80	18.14	no	GBM	methylated	100%	0	100	11
38	M	55	R frontal	70	189.29	no	GBM	unmethylated	90.3%	18.44	70	5
39	M	69	R temporal	80	88.71	no	GBM	methylated	100%	0	90	29
40	F	61	R thalamus	80	81.39	yes	GBM	unmethylated	95.7%	3.51	50	9
41	M	65	L fronto-insular	80	52.59	awake	GBM	unmethylated	100%	0	70	23
42	F	30	R fronto-temporo-insular	60	113.51	no	GBM	methylated	100%	0	50	10
43	M	80	R parietal	90	15.08	no	GBM	unmethylated	100%	0	90	14
44	F	48	L frontal	90	73.19	yes	GBM	methylated	94.4%	4.07	80	4
45	F	61	R parietal	90	12.22	no	GBM	methylated	100%	0	90	27
46	M	56	R frontal	70	176.21	yes	GBM	methylated	100%	0	80	13
47	M	58	L temporo-insular	80	137.65	yes	GBM	unmethylated	100%	0	30	16
48	M	36	R temporal	90	8.39	no	GBM	unmethylated	100%	0	90	14
49	F	70	L parieto-occipital	80	83.05	no	GBM	unmethylated	100%	0	80	9
50	F	72	L fronto-temporal	80	18.42	no	GBM	methylated	100%	0	90	16
51	F	68	L fronto-parietal	60	42.86	yes	GBM	methylated	100%	0	40	alive (39)
52	M	63	L parieto-occipital	90	66.57	no	GBM	unmethylated	100%	0	80	11
53	M	61	R temporal	80	235.64	no	GBM	methylated	99.4%	1.49	90	alive (38)
54	F	64	L parietal	90	27.79	no	GBM	unmethylated	100%	0	90	29
55	M	66	L parietal	80	86.27	yes	GBM	methylated	100%	0	70	alive (37)
56	M	68	L frontal	90	29.51	yes	GBM	unmethylated	100%	0	80	18
57	M	57	R temporal	100	44.57	no	GBM	unmethylated	100%	0	90	16
58	M	54	R fronto-temporal	90	9.65	no	GBM	unmethylated	98.2%	0.17	90	8
59	M	49	R frontal	90	39.19	no	GBM	methylated	100%	0	90	alive (33)
60	M	38	L fronto-parietal	80	61.28	yes	GBM	methylated	100%	0	90	alive (32)
61	M	56	R parieto-occipital	80	21.44	no	GBM	methylated	96.1%	0.83	80	20
62	M	49	L temporo-occipital	50	31.62	no	GBM	unmethylated	100%	0	70	alive (30)
63	F	71	L temporal	80	80.39	no	GBM	methylated	100%	0	80	alive (29)
64	M	51	R thalamic	80	42.17	yes	GBM	methylated	100%	0	50	15
65	F	65	L temporo-occipital	90	2.58	awake	GBM	methylated	100%	0	90	25
66	M	52	R temporal	70	186.45	no	GBM	unmethylated	100%	0	80	19
67	M	66	L temporo-parietal	80	65.53	no	GBM	unmethylated	100%	0	90	13
68	M	60	L temporal	90	1.98	awake	GBM	methylated	100%	0	90	alive (25)
69	F	73	R occipital	70	91.39	no	GBM	methylated	98.7%	1.17	80	15
70	M	69	R parieto-occipital	80	45.65	no	GBM	methylated	100%	0	80	alive (23)
71	M	55	CC (L splenium)	90	1.75	no	GBM	methylated	100%	0	90	14
72	F	77	R parietal	90	53.01	no	GBM	methylated	100%	0	90	alive (22)
73	M	52	R temporo-parietal	80	334.69	yes	GBM	unmethylated	93.2%	22.76	70	10
74	M	62	L parietal	90	9.05	no	GBM	methylated	100%	0	90	alive (19)
75	F	61	R temporal	60	145.09	no	GBM	methylated	100%	0	90	13
76	M	82	R temporal	80	150.05	no	GBM	unmethylated	100%	0	80	10
77	F	73	L temporal	80	77.09	no	GBM	methylated	98.4%	1.25	80	15
78	M	54	R temporo-parietal	100	70.05	no	GBM	unmethylated	100%	0	90	14
79	F	44	R cerebellar	90	6.38	no	GBM	methylated	100%	0	100	alive (16)
80	F	60	L frontal	100	41.37	no	GBM	methylated	100%	0	100	15
81	M	73	R frontal	90	18.71	yes	GBM	methylated	100%	0	80	alive (13)
82	F	47	L temporo-occipital	80	32.71	no	GBM	unmethylated	100%	0	90	6
83	M	63	L temporal	70	30.54	no	GBM	unmethylated	93.9%	1.84	70	alive (12)
84	M	74	L frontal	80	11.83	yes	GBM	unmethylated	100%	0	60	alive (11)
85	M	45	R frontal	100	145.89	yes	GBM	methylated	100%	0	60	alive (9)
86	M	73	L temporal	90	18.39	no	GBM	methylated	95.9%	0.74	90	alive (8)
87	M	53	L fronto-parietal	80	88.41	yes	GBM	unmethylated	97.5%	2.23	80	alive (7)
88	M	66	R frontal	60	18.09	yes	GBM	unmethylated	100%	0	40	alive (6)
89	M	60	L temporo-occipital	80	91.19	no	GBM	methylated	100%	0	70	alive (6)
90	M	43	R temporal	90	63.62	yes	GBM	unmethylated	100%	0	90	alive (6)
91	F	58	L frontal	80	96.64	yes	GBM	unmethylated	100%	0	80	alive (5)
92	F	61	L frontal	90	47.74	no	GBM	methylated	100%	0	90	alive (4)
93	F	70	R temporal	90	89.13	yes	GBM	unmethylated	96.7%	2.93	90	alive (4)

R, right; L, left.

## Data Availability

The data presented in this study are available in the present article. Further data on the review search or data regarding the clinical cases are available upon request to the corresponding author (F.A.).

## Supplementary Materials

The following supporting information can be downloaded at: https://www.mdpi.com/article/10.3390/jcm12010178/s1, Video S1: Fluorescein-guided resection of a right frontomesial glioblastoma (Patient n.88).
